# Effects of Chemical Composition on Anaerobic Digestion Kinetics of Sugar Beet Pulp: Gompertz and Two-Fraction Kinetic Modelling

**DOI:** 10.3390/molecules31111975

**Published:** 2026-06-05

**Authors:** Krzysztof Pilarski, Agnieszka A. Pilarska, Piotr Boniecki, Karol Durczak, Piotr Sołowiej

**Affiliations:** 1 Department of Biosystems Engineering, Poznań University of Life Sciences, Wojska Polskiego 50, 60-627 Poznań, Poland; piotr.boniecki@up.poznan.pl (P.B.); karol.durczak@up.poznan.pl (K.D.); 2 Department of Hydraulic and Sanitary Engineering, Poznań University of Life Sciences, Piątkowska 94A, 60-649 Poznań, Poland; 3 Faculty of Technical Sciences, University of Warmia and Mazury in Olsztyn, Oczapowskiego 2, 10-719 Olsztyn, Poland; pit@uwm.edu.pl

**Keywords:** sugar beet pulp, anaerobic digestion, methane kinetics, chemical composition, kinetic modelling

## Abstract

Anaerobic digestion (AD) of agro-industrial residues supports the green energy transition by converting organic matter into renewable biogas. Sugar beet pulp is a highly fermentable feedstock, although its process response may vary with chemical composition. This study examined how chemical composition affects mesophilic biogas-production kinetics of sugar beet pulp prepared under laboratory conditions from surplus sugar beet roots. The roots represented ten sugar beet varieties (A–J), and the prepared pulp was characterised for pH, dry matter, organic dry matter, mineral composition, and the relative shares of simple sugars, polysaccharides, protein, and fibre. Batch digestion tests were performed at 39 °C for 30 days. Production curves were analysed using complementary kinetic models (modified Gompertz and a two-fraction first-order model) to capture the lag phase and the contributions of rapidly and slowly degradable substrate pools. Biogas yields ranged from 126 to 141 m^3^ Mg^−1^ fresh matter with 50–55% CH_4_, corresponding to 64.3–76.1 m^3^ CH_4_ Mg^−1^ organic dry matter, while organic matter conversion reached 71.2–82.4%. Varieties enriched in simple sugars exhibited a higher share of the fast-degradable fraction and shorter lag phases, indicating faster onset and stronger methane formation. In contrast, higher fibre contents reduced the slow-fraction rate constant and lowered overall conversion, consistent with hydrolysis-limited degradation of the structural carbohydrate matrix. The mineral ion background, particularly K and Na, indicated moderate ionic buffering and stable operation without inhibition. The novelty of this work lies in integrating detailed compositional profiling with dual kinetic modelling to translate chemical fingerprints into tentative process-relevant implications. These implications include feeding strategy, organic loading control and hydraulic retention time selection, and they require further validation in continuous or semi-continuous AD systems.

## 1. Introduction

The energy transition towards a net-zero economy requires renewable energy sources that are at once stable, scalable and embedded within material cycles [1,2]. Anaerobic digestion (AD) of agro-industrial residues has become a central bioprocess in strategies for such low-emission energy systems [1]. Sugar beet pulp meets the key criteria for a robust AD feedstock: it is seasonally available in large volumes and is characterised by a high share of readily biodegradable fractions. It can be directed straight to anaerobic digestion to produce biogas, which may then be upgraded to biomethane with a low carbon footprint [3,4]. At the process-chemistry level, this conversion follows the core pathways of methanogenesis and carbohydrate breakdown. For sugar beet pulp, a carbohydrate-rich feedstock, the AD process can be described using a general reaction scheme. Methane is produced mainly via the acetoclastic and hydrogenotrophic pathways, as shown in Equations (1) and (2). The conversion of the sugar fraction can be represented by a generalised stoichiometric balance for a hexose, as shown in Equation (3). For the structural fraction, polysaccharide hydrolysis is a key rate-limiting step, as shown in Equation (4) [5,6].

CH_3_COO^−^ + H^+^ → CH_4_ + CO_2_ (1)

CO_2_ + 4H_2_ → CH_4_ + 2H_2_O (2)

C_6_H_12_O_6_ → 3CO_2_ + 3CH_4_ (3)

(C_6_H_10_O_5_)_n_ + nH_2_O → nC_6_H_12_O_6_ (4)

In chemical terms, sugar beet pulp comprises readily soluble sugars and a structural carbohydrate fraction dominated by pectin, cellulose and hemicellulose, with only a minor lignin contribution [7]. This composition governs both the amount of fermentable carbon and its accessibility. It also affects hydrolysis kinetics, VFA formation and subsequent methanogenic conversion [6,8]. In parallel, the inorganic ion profile, including K^+^, Na^+^, Ca^2+^, Mg^2+^ and trace metals, determines ionic strength and buffering behaviour. It can also modulate enzyme activity, microbial stress tolerance and the tendency towards VFA accumulation [1,9]. Consequently, an integrated view combining detailed chemical composition with kinetic modelling provides a chemistry-based rationale for the observed varietal differences in methane production dynamics. Here, soluble sugars, structural carbohydrate/fibre fractions, protein, and mineral ions were linked to model-derived kinetic descriptors, including lag phase, maximum production rate, and fast/slow degradation constants.

Substrate composition is one of the primary factors determining the course of hydrolysis and the subsequent conversion of VFA to methane [5,6,8]. Simple sugars, polysaccharides, protein and fibre play distinct metabolic and process roles. Their proportions in the feedstock affect VFA formation, conversion rates and the final methane content of the biogas [10,11]. In parallel, the mineral profile including macro-ions and trace elements shapes ionic strength, buffering capacity and the resistance of the microbial community to fluctuations in organic loading. The literature increasingly emphasises that the stability of AD processes depends on a balance between mineral support for the microbiota and the avoidance of toxic ion concentrations [12]. The relatively uniform character of sugar beet pulp in this respect makes it a suitable substrate for studying links between composition and kinetic response.

At the same time, there are still comparatively few studies that systematically combine a detailed chemical characterisation of the substrate with the parameters of kinetic models describing biogas production. The modified Gompertz model remains the most widely used tool for estimating methane potential, maximum production rate and lag phase. Multi-fraction models split the degradation pathway into fast and slow components. They also allow the effect of hydrolysis rate constants on overall conversion to be assessed [13,14]. In recent years, such models have increasingly been employed to support the design of feeding strategies, the optimisation of hydraulic retention time (HRT) and the minimisation of the risk of VFA accumulation [15,16]. However, there is still a lack of integrated studies that jointly consider varietal variability of the substrate, its full physicochemical, organic and mineral profile, and the resulting differences in process parameters.

The relevance of such analyses clearly extends beyond scientific interest. In engineering practice, knowledge of the shares of fast- and slow-biodegradable fractions enables the selection of appropriate operating strategies and informs key design choices, including HRT, mixing conditions, the degree of digestate recirculation and the acceptable range of organic loading rate (OLR) [12,16]. Varietal differences in the dynamics of hydrolysis and methanogenesis translate directly into the productivity and stability of AD installations, as well as into their ability to operate under seasonally variable feeding regimes. These differences may also have technological implications. Rapidly degradable substrates may support shorter HRT and dense, quasi-isochronous dosing, whereas substrates with a higher fibre content may require slightly reduced OLR or some form of hydrolytic support. Under the growing pressure to increase energy efficiency and ensure operational reliability, these relationships take on strategic importance for plant operators [17].

The aim of this work is to provide an in-depth assessment of how sugar beet pulp composition affects anaerobic digestion kinetic parameters, with particular emphasis on biogas-production kinetics and their compositional determinants. The study is based on a comprehensive experimental dataset obtained for ten sugar beet varieties. The analysis covers physicochemical properties, organic and mineral composition and process performance, and combines two complementary kinetic models: the modified Gompertz model and a two-fraction first-order model. By linking detailed compositional profiling with tandem kinetic modelling, the dataset makes it possible to identify how varietal differences translate into process dynamics, stability and overall conversion. It also supports a cautious interpretation of kinetic behaviour as a potential process-relevant descriptor. The results provide a basis for formulating tentative process-related implications. They may also support further validation studies on the processing of homogeneous plant-based substrates within the framework of the green energy transition.

## 2. Materials and Methods

### 2.1. General Characteristics of Sugar Beet Pulp Feedstock

Surplus, unused sugar beet roots intended for laboratory preparation of sugar beet pulp for anaerobic digestion were handled as fresh, high-moisture biomass. The roots represented ten sugar beet cultivars and were delivered from a sugar factory located in the Wielkopolskie Voivodeship, Poland. Prior to analytical determinations and fermentation, subsamples were taken using stainless steel laboratory tools and weighed on an analytical balance (RADWAG AS 220.R2, RADWAG Wagi Elektroniczne, Radom, Poland). The material was stored in airtight polypropylene containers at 4 °C in a laboratory refrigerator (Liebherr MediLine, Liebherr-Hausgeräte GmbH, Ochsenhausen, Germany) and processed within 48 h to minimise compositional changes associated with spontaneous microbial activity.

Sugar beet pulp is widely recognised in the literature as a physically and chemically homogeneous by-product suitable for anaerobic digestion [3]. Reported characteristics include near-neutral pH, usually 6.3–6.9, total solids of about 20–25%, and a high volatile solids share, often 95–97% of TS [6,7]. Electrical conductivity is typically around 1180–1230 μS cm^−1^, indicating a moderate dissolved-ion load [9]. The mineral profile is usually dominated by potassium, with lower contents of calcium, magnesium and sodium, while trace elements such as Fe, Mn, Zn and Cu occur in ranges typical of plant biomass. This macro- and microelement background provides moderate buffering capacity. It also supports anaerobic digestion stability without indicating ionic inhibition [9,18]. These literature ranges were used only as reference characteristics for contextualising the experimentally determined values reported in Section 2.4.

From an energetic perspective, sugar beet pulp is characterised by a high share of readily biodegradable organic matter. Fresh matter typically contains simple sugars, polysaccharides, protein and a relatively low fibre fraction [3,6]. This composition favours rapid conversion of the soluble fraction and may be associated with high maximum biogas or methane production rates (R_m_) and relatively short lag phases (λ) [8]. In the present study, this background was used to support the interpretation of compositional variability among cultivars and its linkage to kinetic parameters obtained from modelling [5,6].

### 2.2. Sample Preparation

Surplus, unused sugar beet roots representing ten sugar beet cultivars were obtained from the sugar factory described in Section 2.1. The roots originated from ten regions of Poland representing light, medium and heavy soils [19]. The beet pulp used in the anaerobic digestion experiment was prepared from these roots under laboratory conditions. After delivery, the material was visually inspected, and damaged or mould-affected roots were excluded. The beets were rinsed with tap water to remove adhering soil, briefly washed with deionised water and dried at room temperature. For each cultivar, the roots were cut into pieces of approximately 20–30 mm and homogenised before milling.

The beets were milled using an SM 100 cutting mill (Retsch GmbH, Haan, Germany) fitted with a 5 mm sieve, so that the prepared material had a maximum particle size of approximately 5 mm. This ensured material uniformity and supported efficient hydrolysis during fermentation [6]. Each sample was assigned a letter code from A to J; the corresponding cultivars are listed in Table 1. The same codes were used in all physicochemical analyses, BMP assays and kinetic modelling. Additional details on post-milling handling are provided in the Supplementary Materials (Section S1).

### 2.3. Fermentation Conditions and Biogas Measurement

**Figure 1:**
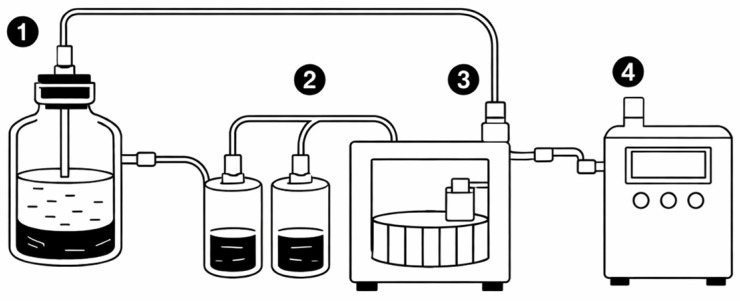
Schematic overview of the BMP reactor and gas measurement line (side view): 1—sealed 2 L anaerobic reactor; 2—gas-tight measurement reservoirs; 3—drum-type gas metre; 4—portable biogas analyser (source: Pilarski et al., 2026 [22]).

Biochemical methane potential (BMP) assays were performed in static, sealed batch reactors in accordance with German DIN/VDI guidance for biomass testing, historically DIN 38414-S8 and currently the widely applied VDI 4630 [20,21]. Fermentation was carried out in 2 L anaerobic reactors operated without substrate feeding, under constant temperature, and without liquid exchange. The inoculum used in the BMP assays consisted of stable anaerobic digestate (active methanogenic inoculum) collected from an operating agricultural biogas plant [4,12]. The only gas handling step was the controlled transfer of produced biogas to the measurement line. Each experimental variant was conducted in triplicate. In each experimental series, inoculum blanks and a positive control were included in the same number of replicates to enable background correction and to validate inoculum activity and test performance [4]. A schematic overview of the experimental setup is presented in Figure 1.

Reactors were charged with inoculum and substrate so that the liquid phase occupied approximately 70–75% of the reactor volume, leaving headspace for gas accumulation. Borosilicate glass reactors (DURAN^®^, DWK Life Sciences, Mainz, Germany) were used and sealed with butyl rubber stoppers and aluminium crimp seals (Sigma-Aldrich, Merck KGaA, Darmstadt, Germany). Prior to each series, leak-tightness of the stopper–crimp–tubing connections was verified to minimise the risk of gas losses. Before incubation, oxygen was removed by flushing the headspace with an N_2_/CO_2_ (80/20, *v*/*v*) mixture. The reactors were then sealed immediately. The flushing gas was supplied by Air Products Sp. z o.o. (Warsaw, Poland). Temperature was maintained at a constant mesophilic level of 39 °C using a forced-air circulation incubator (Binder BD, BINDER GmbH, Tuttlingen, Germany). Reactors were operated without continuous mixing, with the option of gentle rocking when required. Rocking was provided by a rocking shaker (Edmund Bühler SM-30, Edmund Bühler GmbH, Bodelshausen, Germany) operated at low amplitude to support gas balancing while limiting handling-related contamination.

Prior to BMP assays, the inoculum was pre-incubated for 3–7 days at the test temperature without substrate addition until daily biogas production decreased to background levels. The dry matter (DM) and volatile solids (VSs, organic dry matter) contents of the inoculum were determined gravimetrically, analogously to the substrate. Pre-incubation stabilised the methanogenic background and reduced the influence of residual inoculum biodegradability on BMP profiles. The substrate-to-inoculum ratio was set to 0.3 g VS_substrate g^−1^ VS_inoculum to limit the risk of acidification and ammonium-related inhibition [9,18]. Substrate and inoculum doses were calculated from the measured VS contents. Test duration was defined by production stabilisation, i.e., until daily biogas production declined to background. Stabilisation was defined as a decrease in daily production to the inoculum blank level. This condition had to be maintained over subsequent days to ensure closure of cumulative BMP curves and robust kinetic parameter estimation [4].

Daily biogas volume was determined from calibrated, gas-tight measurement reservoirs connected to the reactors via tubing (see Figure 1). The readings were recorded using a drum-type gas metre (Ritter Drum-type Gas Metre, Ritter Apparatebau GmbH & Co. KG, Bochum, Germany). Biogas composition (CH_4_, CO_2_, O_2_, and H_2_S in control mode) was determined using a portable biogas analyser (4) (Geotech GA5000, Geotech, Coventry, UK), serviced and verified by Tusnovics Instruments Sp. z o.o. (Kraków, Poland). Measurements followed the manufacturer’s instructions after stabilisation of analyser readings for each sampled gas stream. CH_4_ and CO_2_ were measured by non-dispersive infrared (NDIR) absorption, whereas O_2_ was measured using an electrochemical sensor, in line with the device specification. The analyser was calibrated with certified reference gases prior to each series and periodically during experiments using at least a two-point (zero/span) procedure. The gases were supplied by Air Products and Chemicals Inc. (Allentown, PA, USA).

### 2.4. Chemical Characterisation and Physicochemical Analysis of Sugar Beet Samples

A compositional fingerprint of each cultivar was established by combining physicochemical analysis, carbohydrate profiling, structural fraction analysis and mineral composition [3,6,7]. Samples were homogenised before analysis. Determinations were performed at least in duplicate, and results were reported as mean values. The analysed parameters included pH, total solids (TSs), volatile solids (VSs), electrical conductivity (EC), macro- and microelement composition, sugar content, polysaccharides, protein and fibre fractions.

The analyses were performed using standard analytical procedures. The pH was measured potentiometrically according to ISO 10523:2008 [23], TS was determined by drying at 105 °C, VS by loss on ignition at 550 °C, and EC conductometrically according to ISO 7888:1985 [24]. Mineral composition, including Fe, Zn, Mn, Cu, Ca, Mg, K and Na, was determined using AAS and ICP-OES after wet mineralisation. Sugar content was determined polarimetrically, polysaccharide hydrolysis products were quantified by HPLC, protein content was determined using the Kjeldahl method with a nitrogen-to-protein conversion factor of 6.25, and fibre fractions, including crude fibre, NDF, ADF and lignin, were determined using detergent fibre analysis.

Detailed instrument specifications, manufacturers, analytical standards and procedural details are provided in the Supplementary Materials (Section S2, Table S1).

### 2.5. Kinetic Modelling of Anaerobic DigestionR

Methane production kinetics from sugar beet pulp were described using two complementary models. The first was the modified Gompertz model, which was applied to cumulative methane production curves. The second was a two-fraction first-order model, which separated the methane potential into rapidly and slowly biodegradable fractions [6,10]. Both models were fitted to the cumulative methane data obtained in batch anaerobic digestion tests [4,10]. Before model fitting, methane volumes were calculated from measured biogas volumes and the corresponding CH_4_ concentrations. Cumulative methane curves were then expressed in the same units as those used in the Results section to ensure direct comparability among cultivars.

The modified Gompertz model was used in the following form (5):

(5) $$M\left(t\right)=P\;exp\left\{-exp\left[\frac{R_{m}e}{P}\left(\lambda -t\right)+1\right]\right\}$$

where

M(t)—cumulative volume of methane produced at time t [m^3^ CH_4_ Mg^−1^ TS], with TS denoting total solids;

P—asymptotic methane yield, interpreted as the biochemical methane potential (BMP) of the substrate [m^3^ CH_4_ Mg^−1^ TS];

R_m_—maximum methane production rate, i.e., the highest slope of the M(t) curve [m^3^ CH_4_ Mg^−1^ TS d^−1^];

λ—lag phase duration, representing the adaptation period before intensive methane formation begins [d];

e—is the base of the natural logarithm (e ≈ 2.71828);

t—fermentation time [d].

The two-fraction first-order model was written as (6)

(6) $$M\left(t\right)=\{\begin{array}{cc} 0, & t\leq r,\\ P\;\left[f\left(1-e^{-k_{1}\left(t-r\right)}\right)+\left(1-f\right)\left(1-e^{-k_{2}\left(t-r\right)}\right)\right], & t>r \end{array}$$

where

M(t)—cumulative volume of methane produced at time t [m^3^ CH_4_ Mg^−1^ TS];

P—overall methane potential of the substrate (identical to the BMP parameter) [m^3^ CH_4_ Mg^−1^ TS];

f (0,1)—share of the fast biodegradable fraction (–);

k_1_ > 0—first-order rate constant for the fast fraction [d^−1^];

k_2_ > 0—first-order rate constant for the slow fraction [d^−1^];

r—time delay before the onset of active degradation [d].

In this formulation, the term f $\left(1-e^{-k_{1}\left(t-r\right)}\right)$ describes the rapid conversion of readily degradable organic matter, mainly soluble sugars, whereas $\left(1-f\right)\left(1-e^{-k_{2}\left(t-r\right)}\right)$ represents the slower degradation of more recalcitrant components such as structural polysaccharides [5,6,10]. The model provides the BMP value, the total methane potential, and the relative contribution and dynamics of the fast and slow fractions. During optimisation, physically meaningful constraints were applied to the estimated parameters (P > 0, R_m_ > 0, λ ≥ 0, 0 < f < 1, k_1_ > 0, k_2_ > 0 and r ≥ 0). Initial parameter values were selected from the experimental curves, using the observed asymptote for P, the maximum incremental slope for R_m_ and the approximate onset time for λ or r.

Model parameters (P, R_m_, λ, f, k_1_, k_2_, and r) were estimated by non-linear least-squares regression in Statistica 13.3 (TIBCO Software Inc., Palo Alto, CA, USA) using the dedicated curve-fitting routines. Parameter estimation was based on minimisation of the sum of squared residuals between measured and model-predicted cumulative methane yields. The optimisation was performed iteratively until successive runs produced negligible changes in the objective function and parameter estimates. Fit quality was assessed using the coefficient of determination (R^2^) and the root mean square error (RMSE). RMSE was calculated as the square root of the mean squared residuals over all fitted time points. The resulting kinetic parameters were then interpreted in relation to the analytically determined physicochemical and chemical characteristics of the sugar beet samples [10].

The applied modelling framework allowed the methane production curves to be described as a function of time and enabled quantitative separation of the fast and slow biodegradable fractions. The estimated parameters were used to interpret differences among cultivars and to assess the practical implications of substrate composition for process performance. In engineering terms, these parameters can support decisions related to hydraulic retention time, feeding strategy, and organic loading rate (OLR, kg organic dry matter m^−3^ d^−1^), as well as evaluation of potential process modifications such as pre-hydrolysis or two-stage digestion [10,12,18].

### 2.6. Experimental Design and Data Analysis

The study applied a comparative design in which all BMP assays were performed under identical operating conditions to ensure direct comparability between cultivars [4]. Replicate BMP results were used to estimate experimental variability, while analytical and instrumental uncertainty components were also considered when interpreting cultivar differences. Data interpretation emphasised effect magnitudes, analytical uncertainty ranges and model-based kinetic descriptors rather than formal hypothesis testing between cultivars. This approach is consistent with metrological practice in analytical chemistry, where measurement uncertainty is used to assess whether observed numerical differences are analytically meaningful [25]. Accordingly, differences between cultivars were discussed only when they were meaningful in relation to the estimated measurement uncertainty and relevant to process assessment.

Relationships between routine substrate indicators and kinetic parameters were explored using descriptive trend analysis and regression- or correlation-based approaches. Model performance was evaluated using goodness-of-fit diagnostics from non-linear least-squares fitting, including R^2^ and RMSE [10]. The degree of organic matter conversion was calculated from the volatile solids (VSs, organic dry matter) balance as the relative decrease in substrate VSs after digestion, with the inoculum blank used to correct for residual organic matter originating from the inoculum.

Measurement uncertainty estimation followed recommendations for laboratory-scale biogas measurements reported in the literature and general guidelines for uncertainty evaluation in analytical measurement [26]. Manufacturer instrument specifications were considered when defining uncertainty components. Repeated readings were used to reduce random error associated with gas volume readings and biogas composition measurements.

## 3. Results

### 3.1. Basic Physicochemical Parameters

Assessment of basic physicochemical parameters provides a foundation for interpreting performance, as pH, TSs, VSs and EC directly define the microbial environment and may affect process stability and gas production kinetics. In this context, Table 2 presents these parameters for samples A–J, corresponding to the analysed sugar beet cultivars, together with the associated measurement uncertainties.

The following cultivars were examined: Janosik, Melodia, Ulla, Mecenas, Olson, Polmar, Tur, Attut, Bryza, and Wojownik (see Table 1). For each sample, pH, TS, VS, and electrical conductivity were determined together with measurement uncertainties. The pH values ranged from 6.3 (sample I, Bryza) to 6.9 (sample F, Polmar), indicating a slightly acidic to near-neutral environment favourable for methanogenic fermentation, for which the optimal pH generally lies within 6.5–7.8 [12,18]. The narrow pH range reduces the likelihood of initial inhibition of methanogenic microbiota. It also supports comparison of kinetic results among cultivars, because pH was not a major differentiating factor during start-up.

Total solids content ranged from 21.6% to 23.1%, with the lowest value observed for sample H (Attut) and the highest for sample C (Ulla). Differences in TS among cultivars may reflect inherent biological traits and cultivation conditions [19]. The mean uncertainty for TS was approximately ±0.19%. Volatile solids ranged from 94.9% (sample C, Ulla) to 97.1% (sample D, Mecenas), expressed as the share of VSs in TSs. The high proportion of VSs within TSs indicates a substantial share of biodegradable matter, which is relevant for biogas potential [17]. The uncertainty for VS was ±0.83%. The consistently high VS fraction suggests that differences in gas yields were more likely related to organic matter quality than to mineral dilution. This includes the balance between rapidly and slowly biodegradable fractions.

Electrical conductivity ranged from 1180 to 1230 μS/cm, indicating a moderate ionic content in the aqueous extract [10]. The lowest conductivity was observed for samples C, E, and G (Ulla, Olson, and Tur), whereas the highest values occurred for samples D and I (Mecenas and Bryza). Conductivity can serve as an indicator of mineral salts and ionic strength, which may influence fermentation performance [9]. Within the observed range, conductivity does not indicate a risk of salinity stress or ionic toxicity. Moderate ionic strength may support process stability by contributing to buffering capacity and ionic transport. These factors facilitate the conversion of volatile fatty acids to methane. Overall, Table 2 enables comparison of key physicochemical characteristics across cultivars. Ulla (C) showed the highest TS, whereas Mecenas (D) exhibited the highest VS share in TSs, which may indicate favourable substrate attributes for biological conversion processes, including anaerobic digestion [27]. The stability of pH and conductivity across samples indicates a broadly uniform substrate matrix [28].

These physicochemical results indicate that all cultivars provided suitable initial conditions for mesophilic anaerobic digestion. The narrow pH range, high VS content and moderate conductivity show that the samples did not differ mainly in terms of basic process constraints. Therefore, the later differences in methane production were probably not controlled by pH, organic matter availability or ionic stress. They should instead be interpreted mainly through differences in substrate accessibility and in the balance between readily degradable and structurally bound organic fractions [6,10,18].

### 3.2. Mineral Composition

The analysis was next extended to mineral composition. The levels of macroelements and trace elements affect ionic strength, buffering capacity and the availability of enzymatic cofactors that are relevant to the biochemical steps of anaerobic digestion. In this context, Table 3 and Table 4 present the contents of macroelements (Ca, Mg, K and Na) and microelements (Fe, Mn, Zn and Cu), expressed per 1 kg of total solids for cultivars A–J, together with the absolute measurement uncertainties.

All results are presented with absolute uncertainty. For macroelements, uncertainty was approximately ±0.056–0.061 g kg^−1^ for Mg and ±0.14–0.15 g kg^−1^ for Ca, whereas for K, it was ±0.39–0.44 g kg^−1^. For trace elements, uncertainty was ±7.5–8.1 mg kg^−1^ for Fe, ±2.0–2.2 mg kg^−1^ for Mn, ±2.9–3.2 mg kg^−1^ for Zn, and ±0.55–0.62 mg kg^−1^ for Cu. For Na, uncertainty ranged from ±6.9 to ±7.4 mg kg^−1^. The mean macroelement contents across the full dataset were Ca = 3.18 ± 0.06 g kg^−1^ TS (SD), Mg = 1.267 ± 0.019 g kg^−1^ TS, and K = 9.11 ± 0.38 g kg^−1^ TS. The Ca range of 3.10–3.28 g kg^−1^ was comparable to the uncertainty of a single determination. Therefore, differences between most cultivars were subtle, although J showed the highest value and B the lowest. For Mg, the observed spread of 1.24–1.30 g kg^−1^ was smaller than the analytical uncertainty. Therefore, inter-cultivar differences should be treated as methodologically insignificant. A different pattern was observed for potassium, because the range of 8.5–9.6 g kg^−1^ clearly exceeded analytical uncertainty. The extreme values therefore indicate genuine differentiation between cultivars. Part of this differentiation may also reflect the regional origin of the roots and the associated soil conditions, because the analysed cultivars originated from areas representing light, medium and heavy soils [19]. Therefore, the mineral profile should be interpreted as the combined effect of cultivar-related properties and source-material conditions.

Variation in potassium is relevant when considered alongside electrical conductivity in Table 2. Cultivars with elevated K, in particular D, I, and J, also belonged to the group with relatively higher conductivity. This indicates that the EC signal mainly reflects the load of macro-cations in the aqueous extract. K^+^ was the dominant contributor, while Na^+^, Ca^2+^, and Mg^2+^ contributed to a lesser extent. At the same time, EC is also influenced by accompanying anions and other ash constituents. Therefore, the relationship between K and EC is moderate and should not be interpreted as fully deterministic. For trace elements, the mean contents were Fe = 119.3 ± 2.5 mg kg^−1^ TS, Mn = 31.9 ± 0.84 mg kg^−1^ TS, Zn = 46.74 ± 1.45 mg kg^−1^ TS, and Cu = 9.02 ± 0.32 mg kg^−1^ TS, whereas Na averaged 109.1 ± 2.6 mg kg^−1^ TS. The standard deviations were smaller than the uncertainties of individual determinations. This suggests that differences between most cultivars for Fe, Mn, Zn, and Cu were below the practical resolution of the method. Exceptions were limited to the most contrasting comparisons, where the Cu difference between G and A exceeded the uncertainty range, and the Na difference between J and A was close to the uncertainty. When Table 2 is considered alongside Table 3 and Table 4, cultivars with elevated K also show higher conductivity, while maintaining favourable pH and a high organic fraction. In practical terms, this indicates a broadly uniform and predictable feedstock profile with moderate variability in ionic load, which supports stable biotechnological operation and interpretation of process measurements without the need for corrections beyond standard buffering practice and moisture control [29].

From a process perspective, the mineral profile indicates that the analysed pulp samples provided a supportive ionic background for anaerobic digestion rather than a limiting factor. The relatively stable concentrations of Ca, Mg and trace elements suggest sufficient micronutrient availability for anaerobic conversion, while the greater variability of K helps explain part of the conductivity differences among cultivars. Because the mineral ranges were moderate, they should be interpreted mainly as stabilising or modulating factors, not as primary drivers of methane yield. The main process-relevant role of the mineral fraction was therefore to support buffering, ionic transport and enzymatic activity, whereas differences in biodegradation behaviour were more strongly linked to the organic profile described in Table 5 [9,12,18].

### 3.3. Organic Profile of Fresh Matter

Defining the organic profile of fresh matter provides a chemical basis for interpreting differences in anaerobic digestion performance. The proportions of rapidly and slowly biodegradable fractions affect the hydrolysis rate and the subsequent methane-production pattern. In this context, Table 5 reports the contents of simple sugars, polysaccharides, protein and fibre in fresh matter for samples A–J. These data were used to interpret differences in process kinetics and organic matter conversion between cultivars.

The fresh matter (FM) was dominated by readily fermentable simple sugars (16.4–20.1% FM, uncertainty of ±1.02–1.25 percentage points). Polysaccharides were present at 6.1–6.7% FM (±0.38–0.42 percentage points). Protein content was low at 1.4–1.8% FM (±0.09–0.11 percentage points). Fibre remained moderate at 1.9–2.4% FM (±0.12–0.15 percentage points). This fraction pattern, combined with pH 6.3–6.9 and conductivity 1180–1230 μS cm^−1^ (Table 2), supports rapid conversion of the soluble fraction and stable methane production [30,31]. The higher sugar contents in I, F, and C provide a larger readily available carbon pool, whereas the higher polysaccharide and fibre contents in G, J, and D increase the share of material requiring hydrolysis before conversion to methane.

The differences observed in Table 5 are particularly important from the perspective of substrate accessibility and biodegradability. Simple sugars represent the fraction that is immediately available for conversion. They therefore directly affect the onset and intensity of methane formation during the early phase of digestion. In contrast, polysaccharides and fibre constitute structurally more complex fractions whose conversion depends on hydrolysis and mass-transfer limitations [5,6]. Even relatively small increases in fibre content may therefore prolong the time required to achieve complete degradation. This explains why cultivars with similar total organic matter content can nevertheless exhibit different methane-production rates and different extents of conversion under the same HRT conditions.

Cultivars with the highest sugar content, Bryza (I), Polmar (F), and Ulla (C), are associated with faster biodegradation. This pattern is consistent with a short lag phase and a high maximum methane production rate [32,33,34]. A slightly higher polysaccharide share can stabilise carbon supply over time. It also increases the demand for adequate HRT to complete hydrolysis of the slower fraction. In this respect, sample E, with a very high biogas volume, and sample D, with the highest methane share, may exhibit favourable gas quality while maintaining only moderate overall conversion. This pattern indicates a hydrolysis constraint in the slowly biodegradable fraction rather than limited utilisation of the readily degradable fraction [35]. The analysed cultivars also differed in the balance between rapidly and slowly biodegradable components, not only in the quantity of individual fractions. Cultivars I, C and partly F combined elevated sugar content with relatively low fibre levels, creating a substrate profile favourable for rapid methane formation and shorter lag phases. By contrast, cultivars G and J exhibited a more structurally constrained profile, in which a larger proportion of carbon was associated with slower hydrolysis pathways. This compositional contrast provides the chemical basis for the kinetic differentiation observed later in both modelling approaches.

Although the protein fraction was small, it contributes nitrogen and supports buffering potential. In combination with a moderate cation background, mainly K^+^ and also Na^+^, Ca^2+^, and Mg^2+^ (see Table 3), the protein fraction can increase resistance to short-term changes in acidity. It can also help maintain pH within the favourable range [36]. Low protein content also limits the risk of ammonia inhibition and foaming, which supports stable methane formation under high carbohydrate loading [37]. Fibre extends the degradation pathway and is linked to slower kinetics. Accordingly, cultivars with relatively higher fibre, such as Tur (G) and Wojownik (J), tend to show slower kinetics and a lower degree of conversion. This pattern is consistent with the fibre data in Table 5 and the kinetic parameters in Table 6. For such profiles, longer HRT or mild hydrolytic support can be considered only as tentative options requiring validation under continuous or semi-continuous operating conditions [38].

These results suggest that simple sugars drive rapid start-up and high short-term methane production rates. Polysaccharides influence the extent of conversion at a given HRT. Protein and mineral elements support process stability. Fibre differentiates the kinetics of the slow fraction. In practical terms, cultivars I, F and C may support more frequent feeding and shorter HRT. Cultivars E and D may benefit from slightly longer HRT or phase separation to improve conversion of the slower fraction. Cultivars G and J require more cautious interpretation because their response is controlled by the slow fraction. For these cultivars, longer HRT, lower OLR or mild hydrolysis support can be considered as tentative options. These suggestions should be treated as tentative because they are based on batch BMP results and require validation in continuous or semi-continuous AD systems.

### 3.4. Energetic Performance in Relation to Substrate Chemistry

The organic composition data in Table 5 were interpreted together with the physicochemical and mineral characteristics in Table 2, Table 3 and Table 4. These data were related to the energetic outcome of anaerobic digestion, expressed as biogas yield and the shares of CH_4_ and CO_2_.

**Figure 2:**
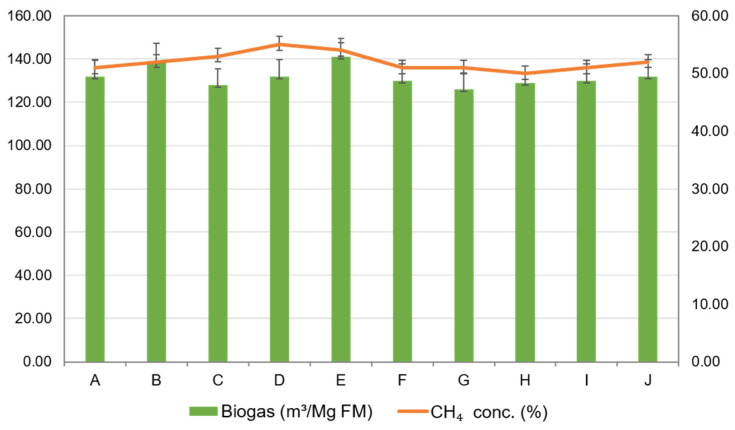
Biogas yield of sugar beet pulp samples expressed per fresh matter (FM): A—Janosik; B—Melodia; C—Ulla; D—Mecenas; E—Olson; F—Polmar; G—Tur; H—Attut; I—Bryza; J—Wojownik. Bars represent mean values; error bars indicate standard deviation based on triplicate BMP assays.

**Figure 3:**
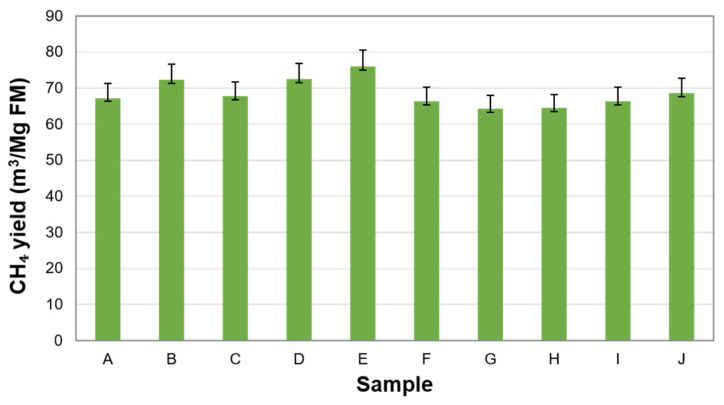
Methane yield of sugar beet pulp samples expressed per fresh matter (FM): A—Janosik; B—Melodia; C—Ulla; D—Mecenas; E—Olson; F—Polmar; G—Tur; H—Attut; I—Bryza; J—Wojownik. FM—fresh matter; CH_4_—methane. Bars represent mean values; error bars indicate standard deviation based on triplicate BMP assays.

Chemical composition, understood as the balance between the soluble and structural fractions and the presence of mineral constituents, affects the availability of methanogenesis precursors. It also affects the rate of their transformation. Therefore, the energetic performance of the analysed samples is presented graphically in Figure 2 and Figure 3. Figure 2 shows biogas yield, whereas Figure 3 shows methane yield, both expressed per fresh matter. These figures provide a direct comparison of chemically driven biodegradability and conversion dynamics across cultivars.

Total biogas yields ranged from 126 to 141 m^3^/Mg fresh matter, which is typical of plant materials with a high share of readily fermentable fractions. The highest yield was obtained for Olson (E, 141 m^3^/Mg fresh matter), whereas the lowest was recorded for Tur (G, 126 m^3^/Mg fresh matter). A difference of 10 to 15 m^3^/Mg fresh matter exceeds the analytical uncertainty and indicates real variation in substrate potential between cultivars. Samples with above-average energetic performance should be interpreted by combining the organic composition data in Table 5 with the biogas and methane yield results shown in Figure 2 and Figure 3. Cultivars I, F and C were characterised by higher simple sugar availability. In contrast, cultivars E, D and B represented high-yielding energetic profiles in terms of methane production per unit of fresh matter. These profiles also occurred under favourable initial conditions, with pH values of 6.3–6.9 and moderate conductivity of about 1.2 mS/cm (Table 2). Such conditions can support rapid VFA conversion to methane [39,40]. As a result, early-phase gas production kinetics were governed mainly by simple sugars and by how evenly they were dispersed within the bioreactor [41,42].

**Figure 4:**
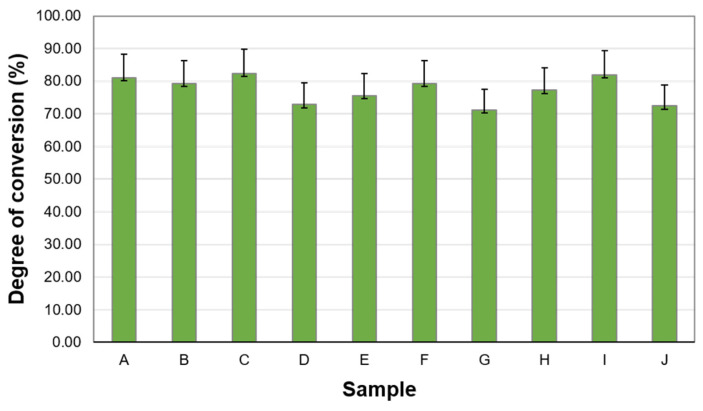
Degree of organic matter conversion in BMP tests for sugar beet pulp samples (A–J): A—Janosik; B—Melodia; C—Ulla; D—Mecenas; E—Olson; F—Polmar; G—Tur; H—Attut; I—Bryza; J—Wojownik. Bars represent mean values; error bars indicate standard deviation based on triplicate BMP assays.

Differences in biogas volume are consistent with the share of the readily available fraction, whereas the energetic value of the gas is largely determined by methane content. Methane content in biogas ranged from 50 to 55%. The highest methane share was obtained for Mecenas (D, 55%), which indicates efficient conversion of VFA to CH_4_ under a balanced organic profile and a supportive mineral background (Table 3 and Table 4: elevated K^+^ and stable levels of Fe, Mn, Zn and Cu) [43,44]. At the same time, the high CH_4_ share for D coincided with moderate overall conversion (Figure 4), which suggests high efficiency of methanogenesis for the readily available fraction and a limitation on the hydrolysis of the slower fraction that includes polysaccharides and fibre. This pattern would be expected to benefit from a slightly longer HRT [45,46]. A similar interpretation applies to Olson (E). High biogas volume and high CH_4_ yield per Mg fresh matter are consistent with dominance of the soluble fraction, whereas the degree of conversion remains constrained by retention time rather than by the methane potential of the biomass [47].

When the structural fraction is relatively higher, process performance depends more strongly on hydrolysis and on the time available to complete conversion. Attut (H, 50% CH_4_) and Tur (G) showed lower methane yield per unit of fresh matter, which is consistent with a higher fibre share and a slightly lower content of simple sugars. This outcome matches the presence of a hydrolytic barrier associated with the slowly biodegradable fraction, which limits precursor availability in the later stage of the process [48]. Quantitative CH_4_ and CO_2_ yields support this trend. Methane yields of about 64 to 76 m^3^/Mg fresh matter increased with the share of simple sugars and with stable process operation. CO_2_ yields of about 59 to 67 m^3^/Mg fresh matter were higher where rapid acidogenesis was not fully balanced by methanogenesis within the applied HRT [49,50]. These relationships also depend on the ionic composition of the medium, which can strengthen or weaken the stability of intermediate conversions. The role of mineral composition is not limited to buffering. Potassium in the range of 8.5 to 9.6 g/kg dry matter and sodium at about 105 to 113 mg/kg, combined with stable pH, support ionic transport and enzyme activity. This may shorten the lag phase and stabilise kinetic parameters in the Gompertz framework when organic overloading does not occur [51,52]. The low protein content of 1.4 to 1.8% fresh matter also reduces the risk of ammonia inhibition and foaming. This lowers the likelihood of a decline in methane content under a high carbohydrate load [53,54].

This composition-to-performance link suggests tentative implications for selecting operating conditions. The results in Figure 2 and Figure 3 provide a preliminary basis for considering feeding strategy and HRT. Cultivars characterised by rapid biodegradation kinetics and high simple sugar availability, particularly I (Bryza), F (Polmar), and C (Ulla), appear compatible with more frequent feeding. They are expected to enter the intensive methane-production phase rapidly, which can help maintain stable VFA levels, limit pH fluctuations and support high CH_4_ yield [55,56]. In contrast, cultivars E (Olson), D (Mecenas), and B (Melodia) should be interpreted primarily as high-yielding energetic profiles, because they produced the highest CH_4_ yields per Mg fresh matter. Cultivars with a more balanced profile, such as D and J, may still provide high methane share and stable operation. A small increase in HRT or low-energy hydrolysis support can be considered to improve overall conversion. Suitable measures include finer comminution, temperature stabilisation and recirculation of the liquid fraction of digestate [57]. Cultivars with a higher fibre share, including G and partly J, need more cautious interpretation because their response is controlled by the slow fraction. For these cultivars, a moderate reduction in OLR or extension of HRT can be considered to raise CH_4_ yield per unit of fresh matter while maintaining process stability. These implications are derived from batch BMP results and should be validated in continuous or semi-continuous AD systems. In all cases, monitoring of VFA to total alkalinity and alkalinity remains beneficial because these indicators are sensitive to dosing errors for sugar-rich substrates [58].

This interpretation is consistent with the preceding sections. Chemical composition defines the balance between the fast and slow fractions. The ionic background supports conversion stability. The findings confirm that process performance depends not only on sugar content but also on the balance between rapidly and slowly biodegradable fractions and on the environmental and mineral conditions [59]. Thus, the apparent difference between the groups I–F–C and E–D–B reflects two different criteria: I–F–C represent rapid degradation behaviour, whereas E–D–B represent the highest energetic output. The data also indicate that cultivars could potentially be combined into feed mixtures in a purposeful way. Simple sugars shape production rate and volume, whereas fibre is converted later when HRT is adequate, and the mineral background and low protein content support the stability of anaerobic digestion [60].

### 3.5. Organic Matter Conversion as a Measure of Degradation Efficiency

In the previous subsection, biogas yield and CH_4_ share were shown to differ among cultivars. However, gas parameters alone do not indicate whether the process exploited the potential of the more slowly biodegradable fraction. To address this, Figure 4 presents the degree of organic matter conversion. This parameter indicates what proportion of organic matter was degraded within the applied retention time. It also helps identify whether the limitation was associated mainly with hydrolysis or with later fermentation steps. The degree of conversion was calculated from the volatile solids (VSs, organic dry matter) balance, using the relative decrease in VSs after digestion and correction for the inoculum blank.

In the analysed set, conversion ranged from 71.2% to 82.4%, with uncertainties of 6.4–7.4 percentage points. These values indicate that the most pronounced contrasts are methodologically robust, with Ulla (C), Bryza (I), and Janosik (A) above 81% and Tur (G), Wojownik (J), and Mecenas (D) at about 71–73%. The high conversion observed for C, I, and A is consistent with their compositional profile reported in Table 5, particularly the higher share of simple sugars and the lower fibre fraction. Higher simple sugar content combined with a low fibre fraction promotes rapid depletion of the readily available fraction and efficient conversion of the VFA stream to methane within the applied HRT. At the same time, favourable physicochemical conditions, including pH of about 6.3–6.9 and conductivity of around 1.2 mS/cm (Table 2), and a balanced level of cations (K, Na, Ca, and Mg in Table 3) stabilise the methanogenic environment and limit pH fluctuations, which supports consistent conversion of soluble carbon [61]. The conversion data also show that high methane production does not necessarily correspond to complete degradation of the organic matrix. In practical terms, cultivars capable of producing high methane volumes over a short period may still leave a larger residual biodegradable fraction in the digestate if hydrolysis of the structural components remains incomplete. This distinction is important because process optimisation based only on gas yield may overestimate the actual stabilisation efficiency of the substrate. Therefore, conversion degree provides complementary information that helps distinguish between rapid methane formation and effective degradation of the total organic fraction [62].

Lower conversion for G and J, as well as D and E, does not indicate inferior feedstock quality but reflects different kinetic constraints. A higher share of polysaccharides and fibre slows hydrolysis and extends the time required for conversion to biogas [62]. This is evident when the yield data in Figure 2 and Figure 3 are considered in relation to the compositional profile reported in Table 5. Olson (E) achieved the highest gas and methane volumes per unit of fresh matter, yet total conversion remained at about 75.6%, indicating that part of the slow fraction was not fully degraded and required a longer HRT. Mecenas (D) represents a clear case of efficient methanogenesis of the readily biodegradable fraction, with 55% CH_4_ and high gas quality, combined with a limitation in the hydrolysis of the slower fraction, which results in a moderate conversion of 72.9%. Tur (G) and Wojownik (J), which had the highest fibre content, form a group of profiles that are stable in operation but require more time to reduce the residual biopolymer load.

The relationship between conversion and biogas yield is not straightforward and should not be interpreted as linear [63]. Ulla (C), which showed the highest conversion, produced a moderate total biogas volume, which is beneficial in practice because it indicates more complete degradation and a reduced risk of residual carbon accumulation in the digestate. In contrast, E and D show a tendency towards rapid degradation of sugars. They produce more gas or a higher CH_4_ share, but they require retention time matched to the slower fraction. This operational distinction between volumetric potential and completeness of degradation should guide strategy selection. If the objective is to maximise m^3^ CH_4_ per Mg of fresh matter over a short horizon, sugar-rich cultivars and time-consistent feeding may be considered. If the aim is to minimise residual load in the digestate and increase overall conversion, greater weight may be given to C- or I-type profiles and to an appropriately selected HRT. These interpretations should be treated as preliminary because they are based on batch BMP results.

The mineral composition is also relevant. Moderately elevated potassium, at 9.1–9.6 g kg^−1^ DM for D, I, and J, and stable conductivity translate into improved buffering and more efficient VFA turnover, which supports high methane share even under a high carbohydrate load [64,65]. At the same time, the relatively uniform levels of Fe, Mn, Zn, and Cu (see Table 4) do not constrain biochemistry, and the low protein fraction, at 1.4–1.8% FM, limits the risk of ammonia inhibition and foaming. This is why the observed differences in conversion are driven mainly by hydrolysis of the slow fraction rather than by toxicity or micronutrient deficiency [66,67]. Thus, the position of a cultivar in the ranking is determined by the biodegradation behaviour of the slowly degradable fraction, the effective hydrolysis rate constant, and the retention time dedicated to that fraction, rather than by background chemistry.

The practical implications should therefore be treated as tentative rather than prescriptive. For rapidly degrading cultivars such as I and F, and partly C, dense isochronous feeding and a shorter HRT appear favourable, as they convert the sugar advantage into high CH_4_ yield without VFA pulses. For productive but not fully closed profiles such as E and D, a moderate increase in HRT by approximately 5–15% can improve conversion. Phase separation with an initial acidogenesis step or recirculation of digestate can also be tested, as these measures may increase conversion without a substantial rise in OPEX (operational expenditure) [68]. For samples G and J with a more balanced fraction profile, two parallel approaches are relevant: a small reduction in OLR and low-energy support of hydrolysis, such as finer comminution and temperature stabilisation [69]. These options require validation in continuous or semi-continuous AD systems before being used as operational recommendations.

The conversion degree in Figure 4 provides a synthetic measure of fermentation quality and, when considered alongside Table 2, Table 3, Table 4 and Table 5, reveals the nature of process limitations. Cultivars C, I, and A show that high conversion aligns with a sugar-dominated substrate and stable environmental conditions. Cultivars E and D demonstrate that high volume and high methane share can be achieved simultaneously, yet completeness of degradation depends on the time available for the more recalcitrant fraction [70,71]. Cultivars G and J indicate that hydrolysis, rather than methanogenesis, often determines the last percentage points of conversion [72]. From an engineering perspective, reactor operation should therefore be interpreted in relation to the shares of the fast and slow fractions and by matching HRT, OLR, and the feeding scheme accordingly [73]. This approach may increase methane yield per unit of feedstock, improve overall conversion, and support operational stability, which is consistent with the requirements of an advanced green transition [74].

### 3.6. Kinetics Based on the Modified Gompertz Model

**Figure 5:**
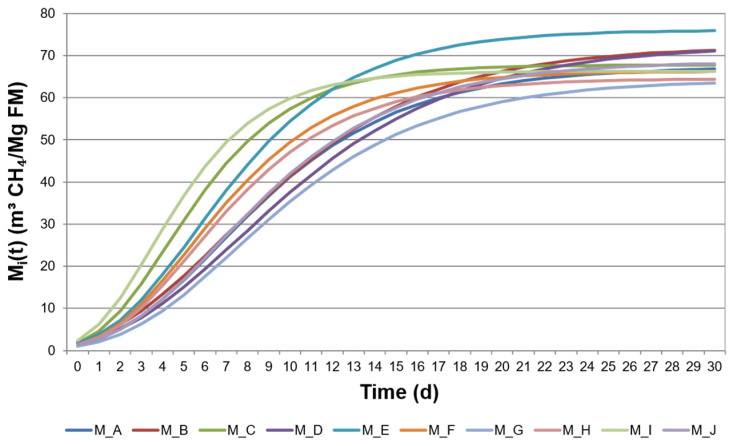
Cumulative methane yield modelled using the modified Gompertz equation (M_i_(t) for cultivars A–J).

With reference to the earlier characterisation of composition, physicochemical properties and biogas/methane production profiles of samples A–J, process dynamics were interpreted using the modified Gompertz model. Figure 5 presents cumulative methane production curves, M(t) (m^3^ CH_4_/Mg FM), fitted with the modified Gompertz model for cultivars A–J. The curves exhibit a sigmoidal pattern comprising a lag phase (λ), a phase of maximum methane production rate (R_m_), and a gradual approach to the asymptote P, defined as the methane potential on a fresh-matter basis. The analysed time window was 0–30 days. Within this period, M(30) was treated as a practical approximation of P, that is, an estimate of the asymptote under a limited observation period. This approach also allowed reliable differentiation of λ and R_m_ among cultivars.

The asymptotic values of P separated the material into distinct performance levels. The highest potentials were observed for E (≈75.9 m^3^ CH_4_/Mg fresh matter), B (≈71.3 m^3^/Mg), and D (≈71.1 m^3^/Mg), followed by J (≈68.1 m^3^/Mg) and C (≈67.8 m^3^/Mg), and then A (≈66.8 m^3^/Mg), cultivar I (Bryza; ≈66.3 m^3^/Mg), and F (≈66.2 m^3^/Mg). The lowest values were recorded for H (≈64.4 m^3^/Mg) and G (≈63.5 m^3^/Mg). Elevated P for E–D–B indicates sustained methane accumulation despite moderate early-stage dynamics. This contrasts with cultivars containing a higher share of readily biodegradable fractions, which approached the plateau earlier at slightly lower P [75]. The lag phase was shortest for cultivar I (Bryza) and C. Their curves departed from near-zero production within days 1–2 and rapidly entered the steep growth region. A short λ was also observed for F and E, while E combined a reduced λ with a high asymptote. Cultivars D, B, A, J, and G exhibited a longer onset, with the period of maximal increments occurring closer to days 6–10. This implies lower R_m_ and a longer time needed to exploit the full methane potential.

The half-saturation time, t_50_, defined as the time required to reach 0.5 P, supported these patterns. Cultivar I (Bryza) reached t_50_ at approximately day 5, C at about days 5.5–5.8, F and H at around day 7, and E also at around day 7. A, B, and J approached t_50_ only at days 8.5–9, and D and G at days 9.5–10. Consistently, the time to reach 95% of the asymptote (t_95_) was shortest for cultivar I (Bryza) and C (≈12–14 days), earlier than for E (≈18 days) and for A/H/B/J (≈21–22 days), and markedly earlier than for D and G (≈24–30 days). Maximum methane production rates (R_m_, interpreted as the model parameter) yielded a clear kinetic ranking: cultivar I (Bryza; ~8.5 m^3^ CH_4_/Mg·d) > C (~7.7) > E (~6.8) > F (~6.3) > H (~6.0) > J (~5.3) ≈ A (~5.1) ≈ B (~4.9) ≈ D (~4.6) ≈ G (~4.6). Interpretation of the Gompertz parameters provides preliminary process-related implications rather than direct operational recommendations. Cultivars with short λ and high R_m_, especially I (Bryza) and C, followed by F and E, appear compatible with frequent and time-consistent feeding and shorter HRT. For high-potential cultivars, such as E, D and B, a moderate extension of HRT can be considered to capture late-stage methane accumulation and to approach the P indicated by M(30). Stable but slower cultivars, including G and partly A, H and J, require more cautious loading and longer residence time. In co-digestion, their use as buffering components should be treated as a tentative option when combined with substrates characterised by short λ. This interpretation requires validation under continuous or semi-continuous operating conditions before being used as process guidance [76]. These kinetic differences indicate that methane performance was controlled not only by final methane potential, but also by the rate at which the readily available fraction was converted. Short λ and high R_m_ for cultivar I (Bryza) and C are consistent with the higher availability of simple sugars and lower structural constraint described in Table 5. In contrast, the slower approach to P for D and G suggests that part of the methane potential was associated with more slowly degradable material, for which hydrolysis rather than methanogenesis was probably the rate-limiting step [6,10,75].

Overall, the Gompertz curves therefore classify the investigated cultivars into three functional groups: kinetically fast (cultivar I (Bryza), C, F), high-potential (E, D, B), and stable but time-demanding (G, with A/H/J showing similar behaviour). This is a descriptive grouping based on batch BMP kinetics. Selection of operating conditions, including HRT, feeding density, and feeding mode, can follow the cultivar position within this framework, but requires process validation. Shorter retention and denser feeding fit profiles with short λ and high R_m_, whereas extended HRT and steady operation fit profiles where completion of P is the main objective. Parameter-informed operation can support both high daily productivity and effective utilisation of the methane potential of sugar beet pulp. However, direct operational use requires validation in continuous or semi-continuous AD systems.

### 3.7. Kinetics Described by the Two-Fraction First-Order Model

**Figure 6:**
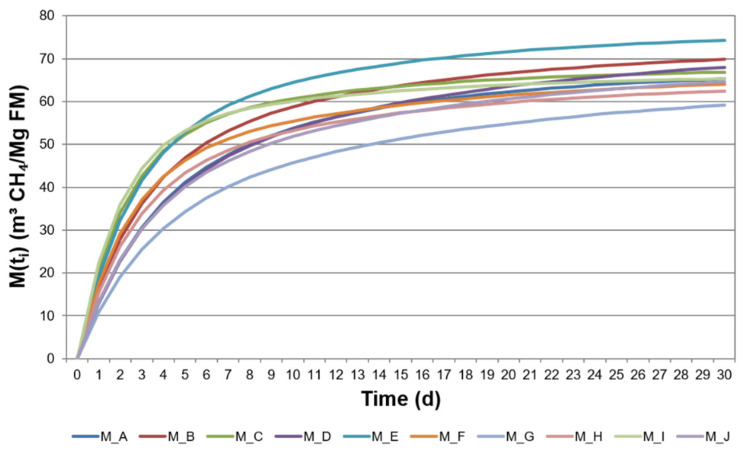
Cumulative methane yield over fermentation time modelled using the two-fraction first-order model (M_i_(t) for cultivars A–J).

To complement the kinetic interpretation based on the modified Gompertz model, a two-fraction first-order model was fitted to the cumulative methane production curves for sugar beet pulp. The results are presented in Figure 6. The plotted trajectories describe M_i_(t) for cultivars A–J and allow estimation of the contributions of the fast and slow fractions using the parameters f, k_1_, and k_2_.

Differences in the contribution of the fast fraction are evident after the first day, as indicated by the M(1)/M(30) ratio. The highest values were obtained for Bryza (I: 22.32/65.32 ≈ 0.34) and Ulla (C: 20.75/66.83 ≈ 0.31), indicating high f and high k_1_. Slightly lower but still elevated fast-fraction shares were observed for Olson (E), Polmar (F), and Attut (H) (approximately 0.25–0.27), whereas Janosik (A), Mecenas (D), Wojownik (J), and Tur (G) were within 0.18–0.21, suggesting lower f and/or lower k_1_. Early-phase dynamics support this interpretation. Over days 1–3, I and C nearly doubled cumulative methane from 22.32 to 44.40 and from 20.75 to 42.70 m^3^ CH_4_/Mg fresh matter, respectively. E and F increased by approximately 19–20 m^3^/Mg. A, D, J, and especially G increased by only 12–15 m^3^/Mg. Expressed as the half-saturation time, t_50_ was approximately 5–6 days for I and C, 6–8 days for E/F/H, 8–9 days for A/B/J, and 9–10 days for D and G. This early separation of I and C confirms that the fast fraction was not only larger, but also more accessible during the first days of digestion. This is consistent with their higher simple sugar content and lower structural constraint shown in Table 5. In contrast, the lower M(1)/M(30) ratios and slower t_50_ values for A, D, J and G indicate that a larger part of their methane potential was distributed over the later phase of the test. The late increments observed for D, G, J and B further show that methane potential was still being released after the main production phase. This pattern is consistent with a larger contribution of structural carbohydrates and fibre-associated material, which require longer conversion time than soluble fractions. The two-fraction model therefore adds information that is not visible from final methane yield alone. It distinguishes rapid substrate availability from delayed methane recovery associated with slower-converting material and provides a basis for interpreting HRT-related implications [6,10,77,78,79,80].

Mid- to late-stage behaviour is governed by closure of the slow fraction, controlled by k_2_. Increments between days 20 and 30 show that I (65.32 − 63.89 = 1.43 m^3^/Mg) and C (66.83 − 65.24 = 1.59 m^3^/Mg) were close to saturation, indicating relatively higher k_2_ and a limited reservoir of the slow fraction (1 − f). Olson (E), despite the highest potential, approached completion at a moderate rate (74.26 − 71.61 = 2.65 m^3^/Mg). Melodia (B) and Wojownik (J) still showed noticeable increments (~3.15 and ~3.96 m^3^/Mg, respectively), consistent with intermediate k_2_ values. The strongest asymptotic inertia was observed for Mecenas (D: 67.97 − 63.50 = 4.47 m^3^/Mg) and Tur (G: 59.18 − 54.83 = 4.35 m^3^/Mg). These profiles indicate a stronger residual contribution of the slow fraction. Where late-stage increments are small, as in I, C, and partly E/F, further extension of HRT would probably provide limited marginal benefit, and stable feeding should remain the priority. Conversely, where clear asymptotic inertia is observed, as in D, G, and also J/B, longer residence time and measures improving the reactivity of the slow fraction remain relevant options. The order of P, based on M(30), was as follows: E ≈ 74.26 > B ≈ 69.79 > D ≈ 67.97 > C ≈ 66.83 > I ≈ 65.32 > A ≈ 65.28 > J ≈ 64.57 > F ≈ 64.04 > H ≈ 62.40 > G ≈ 59.18 m^3^ CH_4_/Mg fresh matter.

Consideration of both potential and kinetics supports three process-relevant profiles. Fast profiles (I, C, and, to a lesser extent, F and partly E) are characterised by high f and k_1_, short t_50_, and limited late-stage gains. This profile is consistent with shorter HRT and time-consistent feeding near the maximum rate. High-potential profiles (E, D, and B) combine moderate k_1_ with high P and a clear need to complete conversion of the slow fraction. In such cases, a moderate HRT extension relative to fast profiles can help exploit the (1 − f) reservoir. Stable but slow profiles (G and partly A and J) are associated with lower f, k_1_, and k_2_. For these profiles, relevant options include a moderate reduction in OLR and gentle support of hydrolysis, such as finer size reduction, stable temperature and controlled digestate recirculation [79,80]. Parameter-based interpretation of differences in f, k_1_, and k_2_ therefore links kinetic behaviour with tentative operating implications. These include high daily productivity at shorter HRT for readily biodegradable fractions, improved conversion for high-potential profiles, and reliable process completion for slower profiles. These implications require validation in continuous or semi-continuous AD systems before being used as operational guidance.

The kinetic parameters obtained from both modelling approaches are summarised in Table 6 to provide a compact overview of the estimated descriptors for all cultivars. Presenting these parameters in a single table facilitates direct comparison of methane potential, kinetic rates, and the relative contribution of fast and slow biodegradable fractions across samples A–J. Goodness-of-fit statistics for the kinetic models are provided in the Supplementary Materials (Table S2).

The table summarises the principal parameters describing methane production dynamics derived from the two modelling approaches. These values provide the quantitative basis for the comparative interpretation of both models presented in the following section.

### 3.8. Comparison of Models and Integrated Interpretation

To integrate the findings obtained using the modified Gompertz model and the two-fraction first-order model, their predictive performance and interpretative value were compared. Both approaches reproduced the methane potential, P, with close agreement, which supports the robustness of the quantitative conclusions. The Gompertz model provides a compact description of process dynamics through P, R_m_, and λ and enables cultivar ranking with respect to start-up behaviour and maximum methane production rate. By contrast, the two-fraction first-order model resolves the same kinetics into a fast fraction (f, k_1_) and a slow fraction (1 − f, k_2_), offering a more mechanistic basis for interpretation. The added value of combining both models is that methane production can be interpreted not only as a final yield, but also as a time-dependent expression of substrate accessibility. In this sense, P describes the amount of recoverable methane within the test window, whereas λ, R_m_, f, k_1_ and k_2_ indicate how quickly this potential becomes available. This distinction is important for sugar beet pulp, because the substrate contains both soluble carbohydrate fractions and more slowly converted structural components. Therefore, model agreement for P supports the reliability of yield estimation, while differences in kinetic descriptors identify the chemical origin of delayed or rapid methane recovery [6,10,13,14].

In the present dataset, the ranking of P was consistent between both approaches and confirmed the highest methane potentials for cultivars E, B, and D, while avoiding the need for an extended symbolic ranking in the text. The detailed numerical comparison is provided in Table 6. The principal differences concerned the explanation of kinetic behaviour. Cultivars D and G showed substantial increments between days 20 and 30 (≈4.5 and ≈4.35 m^3^/Mg, respectively), indicating a considerable contribution of slowly fermenting material. This feature is captured directly by the two-fraction model through a low k_2_ and a larger share of the slow fraction (1 − f). In contrast, cultivars I and C exhibited high M(1)/M(30) values (≈0.34 and ≈0.31), consistent with high f and high k_1_, indicating rapid onset and a limited slow fraction. Such differentiation is relevant for hydraulic retention time (HRT) selection and for process interventions. The Gompertz model indicates the direction of HRT adjustment based on λ and R_m_, but does not identify whether limitation in slow profiles arises from a low fast-fraction share or from slow conversion of the slow fraction. The two-fraction model distinguishes these cases and supports targeted measures aimed at increasing k_2_, such as size reduction, temperature stabilisation, and controlled digestate recirculation, rather than relying solely on longer retention. It also facilitates substrate mixture design through balancing the proportions of fast and slow fractions and selecting feedstocks with favourable f or k_2_ values.

Methane potential assessment remains consistent because P is similar in both approaches. Within the 0–30 day observation window, the two-fraction first-order model provides greater practical utility, as it combines reliable prediction of volume with diagnosis of kinetic constraints (low k_2_ versus low f) and indicates the direction of corrective actions. This is consistent with the process data: biogas yields of 126–141 m^3^/Mg fresh matter, methane content of 50–55% (64.3–76.1 m^3^ CH_4_/Mg fresh matter), and organic matter conversion of 71.2–82.4%. The differences among cultivars indicate distinct substrate-related constraints. Cultivars with higher sugar content (I, F, C) and lower fibre content show a larger readily biodegradable fraction, whereas higher fibre content in G and J limits overall conversion, suggesting a hydrolytic barrier affecting the more slowly degradable fraction [6,7]. Cultivar D, despite the highest CH_4_ share (55%), achieved moderate conversion (≈73%), indicating effective methanogenesis of the readily degradable fraction with incomplete utilisation of complex polymers at the applied HRT [8,9]. Cultivar E maximised gas volume, but a longer retention time can be considered to improve the degree of conversion. This comparison shows that the practical value of a cultivar cannot be judged only by the highest methane potential. A high P value is advantageous when sufficient retention time is available, but it may not translate into the highest short-term productivity if a considerable share of methane is assigned to the slow fraction. Conversely, cultivars with lower P but high f and k_1_ may be more suitable for shorter retention or more frequent feeding, because a larger part of their methane potential is recovered early. The integrated interpretation therefore links cultivar chemistry with operating strategy: sugar-rich profiles support rapid recovery, whereas profiles with greater structural contribution require conditions that improve access to the slow fraction [15,16,77,78,79,80].

Consistency of P between models therefore supports stable quantitative inference, while the resolution provided by f, k_1_, and k_2_ enables closer linkage of observed kinetics with process limitations. Under the analysed conditions, the two-fraction first-order model is consequently more suitable for HRT planning and for selecting process measures aimed at improving organic matter conversion.

## 4. Discussion

The kinetic pattern observed for anaerobic digestion of sugar beet pulp is consistent with the literature on substrates rich in readily hydrolysable carbohydrates. This includes consistent estimates of methane potential (P) obtained with both approaches and clear differentiation between the early and late phases of degradation. Adnane et al. [1] emphasised the importance of agricultural residues in anaerobic digestion systems, and the present results show that such residues may also differ substantially in kinetic behaviour within one feedstock type. Scarlat et al. [2] discussed the bioenergy potential of crop residues, whereas the present study adds a cultivar-level kinetic interpretation for sugar beet pulp. The modified Gompertz model differentiates cultivars with respect to the lag phase (λ) and maximum methane production rate (R_m_). The two-fraction first-order model provides a causal interpretation through the parameters f, k_1_, and k_2_. It helps identify whether the process is limited by conversion of the slow fraction or by depletion of the readily biodegradable fraction. Accordingly, the Gompertz model offers a concise description of the overall trajectory. By contrast, the two-fraction model indicates which component of the organic matrix governs the observed dynamics. This reflects the dual character of sugar beet pulp, which contains both readily available and structural fractions [6,10,13,14].

The value of the dual-model approach is that it separates two effects that are often combined in a single BMP value: the amount of methane that can be recovered and the rate at which this recovery occurs. This distinction is important for sugar beet pulp, because its organic matrix contains both readily soluble carbohydrates and structurally bound polysaccharides. This agrees with Mioduszewska et al. [3], who showed that the biochemical methane potential of sugar beet material depends on substrate condition. Pilarski et al. [4] also highlighted the importance of reliable BMP assessment when comparing organic substrates. A similar methane potential may therefore correspond to different process behaviour when the same carbon pool is released rapidly in one cultivar and gradually in another. Within the 0–30 day profiles, cultivars with short λ and high R_m_ correspond to high f and elevated k_1_. A substantial share of M(t) is then accumulated within days 1–3. This behaviour is typical of substrates with a high proportion of simple sugars and indicates that a larger part of the organic matter was available for rapid conversion during the early phase of the BMP test. In this context, the close agreement in P between the models confirms the consistency of the yield estimate. Differences in λ, R_m_, f, k_1_ and k_2_ reveal how substrate structure controls the time distribution of methane production [6,10,13,14]. This interpretation is more informative than a simple ranking of cultivars by final methane yield, because reactor performance depends on both total recoverable methane and the rate of its release within the selected HRT [15,16].

Cultivars with a higher proportion of structural material, including polysaccharides and fibre, exhibit moderate R_m_, a longer onset, and substantial increments between days 20 and 30. In the two-fraction framework, this corresponds to a relatively lower k_2_ and/or a greater contribution of the slow fraction (1 − f). Chemically, this fraction comprises primarily cell wall components, such as cellulose, hemicelluloses, and pectins. It also includes lignocellulosic complexes, where enzymatic accessibility and diffusion become limiting factors [5,6,7]. This interpretation is consistent with Barakat et al. [5], who showed that xylan structure and lignin–xylan associations affect methane production from C5-sugar-rich material. Ziemiński et al. [6] also demonstrated that hydrothermal pre-treatment of sugar beet pulp can improve methane production, confirming the importance of substrate accessibility. Nowak et al. [7] further showed that process modification with biochar can influence biomethane production from glucose and sugar beet pulp. From an operational perspective, these patterns suggest that extending HRT may yield diminishing marginal gains, whereas measures aimed at increasing effective hydrolysis are more appropriate. Such measures include improved size reduction, temperature stabilisation, controlled recirculation of the liquid fraction, or pre-hydrolytic treatment [15,16,77,78,79,80]. Ionic chemistry of the fermentation medium is also relevant. Divalent cations (Ca^2+^, Mg^2+^) can affect the properties of pectic structures, while alkali ions (K^+^, Na^+^) influence conductivity, mass transfer, and microbial osmotic conditions [9,12,18]. Li et al. [9] showed that salt conditions can affect anaerobic digestion performance. This supports the interpretation that ionic composition should be considered together with substrate chemistry. These operational considerations may also be viewed in a broader engineering context, because agro-industrial substrates such as beet pulp are commonly handled on concrete surfaces and in closed tanks used for methane fermentation. Consequently, the practical optimisation of AD systems should address not only kinetic performance and process stability, but also the durability of construction materials exposed to chemically and biologically aggressive organic media [81].

From the process point of view, the observed cultivar-dependent differences can be explained by the balance between readily accessible organic matter and the structurally bound fraction of the pulp. The D profile, characterised by a high CH_4_ share with moderate overall conversion, indicates efficient utilisation of the readily degradable fraction. The remaining organic matter was probably associated with a slower structural fraction. This supports the use of operating strategies aimed at improving the accessibility of the slow fraction, such as longer retention time or mild hydrolysis support. Process performance was also influenced by the physicochemical and mineral background. Moderate concentrations of Fe, Mn, Zn, Cu, K^+^, Na^+^, Ca^2+^ and Mg^2+^ indicate a supportive ionic environment, provided that inhibitory levels are not exceeded. In the analysed material, this background did not appear to be a limiting factor. Therefore, interpretation of λ, R_m_, f, k_1_ and k_2_ should be linked primarily to the chemical composition of the pulp. This applies especially to the proportions of soluble sugars, polysaccharides and fibre, because these characteristics determine substrate accessibility and the time distribution of methane production [9,12,18,82]. Demirel and Scherer [82] emphasised the importance of trace elements in agricultural biogas systems, and the present results indicate that the analysed mineral background acted mainly as a stabilising factor rather than as a primary yield-limiting variable.

An important aspect of the present findings is the chemical and process-level scope of the applied kinetic interpretation. In the analysed material, the fast fraction was associated mainly with soluble sugars and other readily accessible compounds, whereas the slow fraction reflected the contribution of structural carbohydrates and fibre-associated material. Thus, the fitted parameters should be understood not only as process descriptors, but also as indirect measures of substrate accessibility determined by matrix chemistry. Therefore, references to hydrolysis, acidogenesis, acetogenesis and methanogenic conversion are used here to support process-level interpretation of the observed kinetic patterns. They are not intended to imply direct verification of individual microbial or metabolic pathways. This clarification defines the level at which the obtained parameters should be interpreted and supports a consistent process-oriented reading of the results [5,6,8,10]. Cheah et al. [8] discussed the role of acidogenic conditions in VFA formation, while the present study uses such relationships only at the process-interpretation level to explain kinetic differences derived from substrate chemistry.

The results also show that the highest methane potential is not necessarily the most favourable operational profile. Cultivar E provided the highest methane volume, but its conversion pattern suggests that complete utilisation of the slower fraction depends on sufficient retention time. By contrast, cultivars I and C released a larger share of methane early. This behaviour can be advantageous under shorter HRT or more frequent feeding. Cultivars D and G showed stronger late-stage increments, indicating that a relevant part of their methane potential was associated with slower-converting material. From an engineering perspective, these profiles suggest different tentative control strategies. Sugar-rich and rapidly converting pulps appear more compatible with regular feeding, whereas structurally constrained pulps can benefit from longer residence time, lower OLR or mild support of hydrolysis. These implications should be treated as preliminary because they are based on batch BMP results. Their transfer to process operation requires validation in continuous or semi-continuous AD systems. The results nevertheless indicate that cultivar selection and pulp blending should consider kinetic profile, not only chemical composition or final methane yield [15,16,77,78,79,80]. Pilarska et al. [78] showed that process-supporting materials can improve anaerobic digester performance, while Hupfauf et al. [80] demonstrated that operating conditions can strongly affect biomethanation efficiency. These studies support the relevance of linking substrate profile with process strategy, provided that such links are validated under process-relevant operating conditions.

The main relationships identified between chemical composition and kinetic behaviour were therefore summarised as an exploratory descriptive synthesis. This grouping is not intended as a statistically validated cultivar classification or as direct process guidance. It should be treated as a hypothesis-generating interpretation of batch BMP behaviour. The detailed classification table is provided in the Supplementary Materials (Table S3).

Final methane yield alone is insufficient for process evaluation, because cultivars with similar *p* values may differ in λ, R_m_, f, k_1_ and k_2_. Labatut et al. [75] similarly showed that BMP and biodegradability should be interpreted together for complex organic substrates. In the present study, the exploratory grouping was therefore used only to organise the interpretation of chemical and kinetic patterns, not to define validated operational classes. On this basis, further experimental and application studies should quantify relationships between kinetic parameters (λ, R_m_, f, k_1_, and k_2_) and the chemical profile of the pulp. This should include the sugar fraction, pectins, hemicelluloses and cellulose, together with structural indicators such as insoluble solids content and particle size. Future work should also assess how ionic composition and alkalinity affect process stability under continuous operation. This should be complemented by intervention studies aimed at increasing k_2_ and by evaluation of transferability across a range of HRT and OLR in continuous or semi-continuous systems. Such validation would enable the development of chemically justified criteria for cultivar selection and process conditions to maximise CH_4_ yield and organic matter conversion [15,16,17,18,77,78,79,80,81,82].

## 5. Conclusions

This study showed that sugar beet pulp from different cultivars differed in methane yield, organic matter conversion and digestion kinetics under the applied BMP conditions. The use of the modified Gompertz model and the two-fraction first-order model allowed these differences to be described in terms of methane potential, start-up rate and the relative contribution of rapidly and slowly degradable substrate fractions.

1. Methane production from the tested pulp samples ranged from 126 to 141 m^3^ Mg^−1^ fresh matter, with methane concentrations of 50–55%. Organic matter conversion ranged from 71.2 to 82.4%. These results indicate measurable differences among cultivars under the same experimental conditions.
2. The highest methane potential was observed for cultivar E, followed by B and D, whereas the lowest values were recorded for H and G. Within the 30-day test period, M(30) provided a practical basis for comparing methane potential among the samples.
3. Cultivars I and C showed the fastest process start-up. They had the shortest lag phase, high maximum methane production rates and short t_50_ and t_95_ values. In the two-fraction model, these samples were also characterised by high values of the fast biodegradable fraction and its rate constant.
4. Cultivars D and G showed slower completion of methane production. Their kinetic profiles were associated with a larger contribution of the slow fraction and lower k_2_ values, suggesting that longer retention time could be considered to approach their full methane potential.
5. The relationship between composition and kinetics suggests that a higher share of simple sugars favoured rapid methane production in the early phase of digestion. In contrast, higher contents of structural components, especially polysaccharides and fibre, were associated with slower degradation and lower conversion within the tested period.
6. The mineral background of the pulp samples was moderate and did not indicate conditions likely to limit anaerobic digestion. The observed relationships between composition, methane production and kinetic parameters provide a process-level basis for interpreting differences among cultivars. They may also support preliminary consideration of operating strategies.
7. From the perspective of the sugar beet industry, the results indicate that sugar beet pulp should not be treated only as a uniform by-product. Cultivar-related differences in composition and digestion kinetics may support preliminary decisions on pulp allocation, storage, blending, hydraulic retention time and organic loading rate in biogas plants integrated with sugar factories. Faster-degrading pulp profiles could be considered for shorter-retention or higher-throughput systems. Pulp with a larger slow-degradable fraction may require longer retention time, lower loading or blending with more readily degradable material. These process-related implications should be treated as tentative. They were derived from batch BMP assays and require validation in continuous or semi-continuous AD systems before being used as operational guidance.

## Figures and Tables

**Table 1:** Designation of individual samples.

Beet Cultivar	Sample Designation
Janosik	A
Melodia	B
Ulla	C
Mecenas	D
Olson	E
Polmar	F
Tur	G
Attut	H
Bryza	I
Wojownik	J

**Table 2:** Basic physicochemical parameters of sugar beet pulp samples (cultivars A–J).

Sample	pH (–)	Uncertainty (+/−)	TSs (%)	Uncertainty (+/−)	VSs (%)	Uncertainty (+/−)	Conductivity (μS/cm)	Uncertainty (+/−)
A	6.7	0.060	22.1	0.19	96.4	0.83	1190	10.9
B	6.8	0.061	22.4	0.19	95.6	0.82	1220	11.1
C	6.5	0.058	23.1	0.20	94.9	0.82	1180	10.8
D	6.6	0.059	21.9	0.19	97.1	0.84	1230	11.2
E	6.5	0.058	22.6	0.19	96.2	0.83	1180	10.8
F	6.9	0.062	22.9	0.20	95.8	0.83	1190	10.9
G	6.7	0.060	21.8	0.19	95.9	0.83	1180	10.8
H	6.4	0.057	21.6	0.19	96.5	0.83	1195	10.9
I	6.3	0.056	22.7	0.20	96.1	0.83	1230	11.2
J	6.5	0.058	21.9	0.19	96.6	0.83	1215	11.1

**Table 3:** Contents of selected macroelements in sugar beet dry matter (per 1 kg of total solids) by cultivar.

Sample	Ca (g/kg)	Uncertainty (+/−)	Mg (g/kg)	Uncertainty (+/−)	K (g/kg)	Uncertainty (+/−)	Na (mg/kg)	Uncertainty (+/−)
A	3.20	0.15	1.25	0.057	9.1	0.41	105	6.85
B	3.10	0.14	1.30	0.059	8.5	0.39	110	7.18
C	3.15	0.14	1.28	0.058	8.8	0.40	107	6.98
D	3.25	0.15	1.27	0.058	9.5	0.43	108	7.05
E	3.18	0.14	1.26	0.057	9.2	0.42	106	6.92
F	3.12	0.14	1.29	0.058	8.7	0.39	109	7.11
G	3.22	0.15	1.24	0.056	9.3	0.42	112	7.31
H	3.16	0.14	1.25	0.057	8.9	0.40	110	7.18
I	3.17	0.14	1.27	0.058	9.5	0.43	111	7.24
J	3.28	0.15	1.26	0.057	9.6	0.44	113	7.37

Explanations: Ca—calcium; Mg—magnesium; K—potassium; Na—sodium.

**Table 4:** Contents of selected microelements in sugar beet dry matter (per 1 kg of total solids) by cultivar.

Sample	Fe (mg/kg)	Uncertainty (+/−)	Mn (mg/kg)	Uncertainty (+/−)	Zn (mg/kg)	Uncertainty (+/−)	Cu (mg/kg)	Uncertainty (+/−)
A	120.5	7.86	32.1	2.09	45.2	2.95	8.5	0.55
B	115.3	7.52	30.7	2.00	48.0	3.13	9.1	0.59
C	118.0	7.70	31.5	2.06	44.8	2.92	8.7	0.57
D	122.1	7.97	33.2	2.17	46.5	3.03	9.3	0.61
E	119.8	7.82	31.9	2.08	47.2	3.08	9.0	0.59
F	116.7	7.62	30.9	2.02	45.5	2.97	8.8	0.57
G	121.0	7.90	32.5	2.12	49.1	3.20	9.5	0.62
H	117.5	7.67	31.2	2.04	46.7	3.05	9.0	0.59
I	119.0	7.77	32.0	2.09	45.9	3.00	8.9	0.58
J	123.3	8.05	33.0	2.15	48.5	3.16	9.4	0.61

Explanations: Fe—iron; Mn—manganese; Zn—zinc; Cu—copper.

**Table 5:** Chemical composition of sugar beet in fresh matter (cultivars A–J).

Sample	Simple Sugars (%)	Uncertainty (+/−)	Polysaccharides (%)	Uncertainty (+/−)	Protein (%)	Uncertainty (+/−)	Fibre (%)	Uncertainty (+/−)
A	16.4	1.02	6.2	0.39	1.5	0.09	2.0	0.12
B	17.8	1.11	6.5	0.41	1.6	0.10	2.1	0.13
C	18.9	1.18	6.1	0.38	1.4	0.09	1.9	0.12
D	17.5	1.09	6.7	0.42	1.7	0.11	2.3	0.14
E	18.0	1.12	6.3	0.39	1.6	0.10	2.0	0.12
F	19.3	1.20	6.4	0.40	1.5	0.09	2.2	0.14
G	17.7	1.10	6.6	0.41	1.8	0.11	2.4	0.15
H	18.2	1.13	6.5	0.41	1.5	0.09	2.1	0.13
I	20.1	1.25	6.2	0.39	1.6	0.10	2.0	0.12
J	17.9	1.12	6.6	0.41	1.7	0.11	2.3	0.14

**Table 6:** Kinetic parameters obtained from the modified Gompertz model and the two-fraction first-order model for sugar beet cultivars A–J.

Sample	P (m^3^ CH_4_ Mg^−1^ FM)	R_m_ (m^3^ CH_4_ Mg^−1^ d^−1^)	λ (d)	f	k_1_ (d^−1^)	k_2_ (d^−1^)	r (d)
A	67.30	4.837	1.565	0.570	0.369	0.082	0.75
B	72.30	5.246	1.396	0.621	0.414	0.080	0.33
C	67.80	8.349	0.833	0.735	0.535	0.085	0.58
D	72.60	4.533	1.680	0.556	0.356	0.070	0.91
E	76.10	6.051	1.234	0.654	0.432	0.080	0.25
F	66.30	7.100	1.026	0.667	0.477	0.077	0.83
G	64.30	4.652	1.732	0.534	0.339	0.066	0.50
H	64.50	6.331	1.132	0.649	0.440	0.080	1.00
I	66.30	8.500	0.712	0.759	0.555	0.082	0.41
J	68.60	5.104	1.419	0.605	0.399	0.067	0.66

Explanations: P—methane potential estimated from the modified Gompertz model; R_m_—maximum methane production rate; λ—lag phase; f—share of the fast biodegradable fraction; k_1_ and k_2_—first-order rate constants of the fast and slow fractions; r—time delay before the onset of active degradation.

## Data Availability

The data presented in this study are available from the corresponding author upon reasonable request and subject to confidentiality restrictions. Raw data are not publicly available.

## Supplementary Materials

The following supporting information can be downloaded at: https://www.mdpi.com/article/10.3390/molecules31111975/s1, Section S1: Post-Milling Sample Handling; Table S1: Analytical methods, instrumentation and standards used for physicochemical and chemical characterisation of sugar beet samples; Section S2: Analytical Methods and Instrumentation; Table S2: Goodness-of-fit statistics for kinetic models; Section S3: Kinetic Model Fit Evaluation; Table S3: Exploratory descriptive synthesis of chemical profile, kinetic response and tentative process implications for sugar beet cultivars; Section S4: Exploratory Cultivar Classification.
