# Supplementary material for: Effects of Chemical Composition on Anaerobic Digestion Kinetics of Sugar Beet Pulp: Gompertz and Two-Fraction Kinetic Modelling
Source: Molecules. 2026 Jun 5;31(11):1975. doi: 10.3390/molecules31111975 (PMC13257436; doi:10.3390/molecules31111975)
Supplement: Supplementary file 1 [file molecules-31-01975-s001.zip › molecules-4235008-supplementary.pdf]

Supplementary Materials

Section S1. Post-Milling Sample Handling

Between cultivars, the grinding chamber, rotor and sieve were cleaned with deionised water and wiped with 70% (v/v) ethanol to prevent cross-contamination. The milled material was gently mixed to homogenise the particle-size distribution and transferred to airtight polypropylene containers. Prepared samples were stored at 4 °C until use and were allowed to equilibrate to room temperature before analytical determinations and inoculation. Where required, the material was re-homogenised immediately before weighing and dispensing.

Section S2. Analytical Methods and Instrumentation

Table S1. Analytical methods, instrumentation and standards used for physicochemical and chemical characterisation of sugar beet samples.

**Determined parameter**	**Analytical method**	**Instrument/standard**
pH	Potentiometric measurement	SevenCompact S220 pH meter (Mettler-Toledo GmbH, Greifensee, Switzerland); CPC-505 pH/conductivity meter (Elmetron Sp.j., Zabrze, Poland); ISO 10523:2008
Total solids	Gravimetric drying at 105 °C	Forced-air oven (Memmert GmbH, Schwabach, Germany); ISO 11465:1993
Volatile solids	Loss on ignition at 550 °C	Muffle furnace (Czylok, Jastrzębie-Zdrój, Poland)
Electrical conductivity	Conductometric measurement	CPC-505 conductivity meter (Elmetron Sp.j., Zabrze, Poland); ISO 7888:1985
Fe, Zn, Mn, Cu, Ca, Mg, K, Na	AAS / ICP-OES	ContrAA® or AAnalyst systems; Optima 2100 DV spectrometer (PerkinElmer Inc., Waltham, MA, USA)
Sucrose	Polarimetric determination	Polax 2L polarimeter (Atago Co. Ltd., Tokyo, Japan)
Polysaccharide hydrolysis products (glucose, pentoses, galacturonic acid, phenylpropanoid alcohols)	HPLC analysis	Agilent 1200 Series (Agilent Technologies, Santa Clara, CA, USA) or Shimadzu LC-20 (Shimadzu Corp., Kyoto, Japan)
Total nitrogen	Kjeldahl digestion and distillation	KjelFlex K-360 unit (BÜCHI Labortechnik AG, Flawil, Switzerland)
Crude fibre, NDF, ADF, lignin	Detergent fibre analysis	Fibertec™ system (FOSS Analytical A/S, Hillerød, Denmark)

Section S3. Kinetic Model Fit Evaluation

Goodness-of-fit statistics for the kinetic models are summarised in Table S2. R^2 and RMSE were used to compare how well the modified Gompertz model and the two-fraction first-order model reproduced the methane production curves. Higher R^2 and lower RMSE indicate better agreement between measured and model-predicted values.

Table S2. Goodness-of-fit statistics for kinetic models.

Sample	R^2 Gompertz	RMSE Gompertz	R^2 two-fraction	RMSE two-fraction
A	0.839	7.485	0.864	6.885
B	0.806	8.780	0.800	8.920
C	0.907	6.122	0.879	6.966
D	0.799	8.713	0.867	7.087
E	0.850	8.088	0.857	7.899
F	0.871	7.114	0.863	7.324
G	0.896	5.873	0.872	6.501
H	0.885	6.535	0.885	6.552
I	0.914	5.880	0.886	6.785
J	0.883	6.973	0.858	7.674

Section S4. Exploratory Cultivar Classification

Table S3. Exploratory descriptive synthesis of chemical profile, kinetic response and tentative process implications for sugar beet cultivars.

Profile type	Cultivars	Dominant chemical feature	Kinetic response	Tentative process implication
Fast-degrading	I (Bryza), C (Ulla), F (Polmar)	High simple sugars, low fibre	Short λ, high R_m, high f, high k_1	May support shorter HRT and more frequent, time-consistent feeding
High-potential	E (Olson), D (Mecenas), B (Melodia)	High methane potential with moderate slow-fraction closure	High P, moderate λ/R_m, incomplete late conversion	May benefit from slightly longer HRT to improve slow-fraction conversion
Slow-structural	G (Tur), J (Wojownik), partly A (Janosik) and H (Attut)	Higher fibre and structural fraction	Lower k_2, prolonged late phase	May benefit from hydrolysis support, lower OLR or longer HRT

This table presents an exploratory descriptive synthesis based on batch BMP results. It is not a statistically validated cultivar grouping, and the tentative process implications require validation in continuous or semi-continuous anaerobic digestion systems.
